# Application of BI-EHEC and BI-EPEC bacteriophages to control enterohemorrhagic and enteropathogenic escherichia coli on various food surfaces

**DOI:** 10.1186/s13104-023-06371-6

**Published:** 2023-06-13

**Authors:** Leny Agustina L.A, Diana Elizabeth Waturangi

**Affiliations:** Faculty of Biotechnology, Atma Jaya Catholic University of Indonesia, Jalan Jenderal Sudirman 51 Jakarta, 12930 South Jakarta, Indonesia

**Keywords:** Bacteriophages, Enterohemorrhagic *Escherichia coli* (EHEC), Enteropathogenic *Escherichia coli* (EHEC), Foodborne disease, Biocontrol

## Abstract

**Objectives:**

The purposes of this study were to determine the Efficiency of Plating (EOP) value of Bacteriophage BI-EHEC and BI-EPEC and to evaluate the application of these bacteriophages in reducing population of EHEC and EPEC on various food samples.

**Results:**

In this study, we used bacteriophage BI-EHEC and BI-EPEC, which were isolated from previous study. Both phages were tested with other multiple pathotypes of intestinal pathogenic *E. coli* to determine the efficiency of plating. BI-EHEC had high efficiency toward ETEC with an EOP value of 2.95 but low efficiency toward EHEC with an EOP value of 0.10, while BI-EPEC had high efficiency toward EHEC and ETEC with EOP values of 1.10 and 1.21, respectively. As biocontrol agents, both bacteriophages able to reduce CFU of EHEC and EPEC in several food samples using 1 and 6-days incubation times at 4 $\text{?}$. BI-EHEC reduced the number of EHEC with an overall percentage of bacterial reduction value above 0.13 log_10_, while BI-EPEC reduced number of EPEC with reduction value above 0.33 log_10_.

**Supplementary Information:**

The online version contains supplementary material available at 10.1186/s13104-023-06371-6.

## Introduction

Foodborne disease (FBD) happens after ingesting food that is contaminated with pathogenic microbial or even its toxin, with symptoms such as diarrhea, gastroenteritis, inflammation, and nutrient malabsorption [1]. FBDs are caused by diarrheal disease agents, which are mainly Diarrheagenic *Escherichia coli* (DEC). These bacteria are enterovirulent *E. coli* pathogenic strains that are especially problematic in human clinical medicine and are established as food pathogens that cause diarrhea. Some *E. coli* strains have virulence genes that can cause gastroenteritis in children. The most common pathogenic strains that are involved in foodborne illnesses are Enterotoxigenic *E. coli* (ETEC), Enteropathogenic *E. coli* (EPEC), Enterohemorrhagic *E. coli* (EHEC), and Enteroinvasive *E. coli* (EIEC) [2]. These strains have the competence to acquire virulence factors that qualify them to invade the gastrointestinal tract of the human body and animals, which can cause diseases [3]. In terms of antimicrobial resistance, several *E. coli* strains are resistant to penicillin G (β-lactam) because they produce β-lactamase encoded by the plasmid [4].

EHEC is classified as a pathogenic Shiga toxin-producing *E. coli* (STEC) that can cause human diseases, such as watery diarrhea, bloody diarrhea, and hemolytic uremic syndrome. EHEC is known to produce proteins called adhesins that are useful for adhesion and beneficial for establishment, persistence, and tissue tropism during infection [5]. Virulence factors that are owned by EHEC are Shiga toxins, which are Stx1 and Stx2. Stx can help EHEC adhesion to host epithelial cells by upregulating surface expressions of two receptors which are phosphatidylethanolamine and nucleolin [6]. EHEC produces hemolysin during the infection, which infects the human host cell. It kills target cells by entering the cell membrane, which creates pores and causes cell lysis. EHEC has a low infectious dose at 1 to 100 CFU. At 20 $?$, the population of EHEC increases by > 1 log CFU/g within 24 h and remains constant thereafter. Meanwhile, at 4 $?$, the population of EHEC decreases by a 2 log CFU/g after 5 days of inoculation [7].

EPEC is a noninvasive pathogenic bacterium that causes infantile diarrhea, when enters the gastrointestinal tract, it adheres to the mucosa of the small and large intestines [8]. EPEC is also classified as an extracellular pathogen [9] and attaches to gut epithelial cells and produces bundle-forming pilus (*bfp*). This bacteria secretes virulence factors into the host cell [10] and it is known to be resistant to ampicillin, ticarcillin, cephalosporins, and cotrimoxazole [11]. EPEC infects healthy adults at 10^8^ CFU. These bacteria are mostly found in contaminated foods, such as raw clover sprouts, lettuce, cucumbers, raw meat, dairy products, or contaminated environments [12].

To reduce bacterial contamination, many methods have been carried out, such as disinfection with organic acids, water vapor, and irradiation but these may affect foods’ organoleptic and nutrients [13]. In addition, chemical sanitizers are corrosive and not environmentally friendly [14]. Meanwhile, some strains of pathogenic bacteria are resistant to several antibiotics and bacteriophage have been used nowadays in many fields as an alternative control against various pathogenic bacteria which are resistant to antibiotics [15]. Hence, study on bacteriophage as alternative treatment is required.

Bacteriophage is bacterial viruses that have specifically only infected bacteria with a specific host target. Bacteriophages have genetic material that consists of double-stranded or single-stranded DNA or RNA. Phage infection to a host bacterium is initiated by receptors on the host cell. After adsorption and it injects its genome into the host and takes over much of the host’s metabolism and sets up molecular machinery for the replication and assembly of more bacteriophages [16]. Bacteriophages in the *Myoviridae* family have long and contractile tails. Bacteriophage tails consist of tail fibers, tail spikes, and tail tips, which all function as RBPs to recognize host receptors, such as teichoid, acids, porins, and lipopolysaccharide. The RBPs of tailed phages have a high genetic plasticity which enables them to infect new hosts. The mechanism of bacteriophage infection involves initiating reversible attachment followed by irreversible adsorption and injection of the phage genome into the host cytoplasm. The main receptor in Gram-negative bacteria, such as *E. coli*, is a lipopolysaccharide that appears in both smooth (presence of O-antigens) and rough (absence of O-antigens). Bacteriophages recognize the O-antigen with their tail fibers or tail spike proteins, which hydrolyze the O-antigen to initiate penetration of the tail. Besides lipopolysaccharides, bacteriophages also attach to outer membrane porins, such as OmpC and OmpF [17]. Comprehensive study have been conducted by using cocktail bacteriophage to control pathogenic *E. coli* [18]. As biocontrol agents, bacteriophages have several characteristics that are functional in eliminating bacteria, such as high specificity to target their host, self-replication, self-limiting, adaptable to altered host systems, low inherent toxicity, easy to isolate and propagate, and prolonged shelf life.

## Methods

### Bacterial growth

EHEC, EPEC, and ETEC from US Namru-2 were used in this research as hosts for bacteriophages and artificial contamination assays. All the bacterial strains were stored in 1.0 mL aliquots in 20% (v/v) sterilized glycerol stocks at -80 $$? $$. Bacterial cultures were inoculated onto Luria Bertani (LB) agar plates and incubated at the optimum growth conditions for the bacteria (37 $$?$$) overnight. The LB agar plates were stored at 4 $$?$$ and used as the working culture for the next assays.

### Double agar overlay assay

Bacteriophages BI-EHEC of EHEC and BI-EPEC of EPEC were from previous research and were isolated from beef intestines [19]. Bacteriophage refreshment was carried out with the double agar overlay assay. First, each bacterial strain was grown in LB broth to the mid-log phase with incubation in a water bath shaker at 37 ℃, 120 rpm, for 6–8 h. For the double agar overlay assay, 200 µL of the bacteriophage filtrate from the previous research, 200 µL of the mid-log phase bacterial culture (108 CFU/mL or OD600 = 0.132), 50 µL of 10 mM CaCl2, and 10 µL mM MgSO4 were mixed by vortexing. The mixture was incubated for 20 min at 28 ℃. Following the incubation, the mixture was combined with 5 mL of soft LB agar with 0.6% (w/v) agar and vortexed. After that, the mixture was poured onto the surface of the LB agar plate with 2% (w/v) agar immediately. The agar plate was incubated overnight at 37 ℃. The following day, the plaques were analyzed and classified by morphology [19–22].

### Bacteriophage purification

Bacteriophages were purified by single plaque isolation using a sterile tip. The area around the plaque was stabbed and sucked into the sterile tip (~ 2 µL) using a micropipette. Purified plaques were suspended in LB broth with 200 µL of the mid-log phase bacterial culture and supplemented with 50 µL of CaCl2 10 mM and 10 µL of MgSO4 10 mM, and the mixture was vortexed. After that, the mixture was incubated in a water bath shaker at 37 ℃, 120 rpm, overnight. Following the incubation, the mixture was centrifuged at 7000 x g for 15 min. After centrifugation, the supernatant was filtered through a 0.22 μm pore size membrane filter (HIMEDIA) to obtain a pure bacteriophage lysate. The purified bacteriophage was kept at 4 ℃ with the addition of Ringer Solution (RS) [1:1 (v/v) ratio] as a working solution for the next assays. A 10-fold serial dilution was also performed and plating was done using the double agar overlay assay until the morphologies of all plaques were consistent [19, 20, 23, 24].

### Bacteriophage titer determination

Bacteriophage titer determination was carried out by the double agar overlay assay. The purified bacteriophage stock was serially diluted (10-fold serial dilution) using sterilized Sodium of Magnesium (SM) buffer [0.05 M Tris-HCl, pH 7.5, 0.1 M NaCl, 0.008 M MgSO4, 0.01% gelatine]. For the double agar overlay assay, 200 µL of the diluted bacteriophage solution, 200 µL of the mid-log phase bacterial culture (108 CFU/mL or OD600 = 0.132), 50 µL of CaCl2 10 Mm, and 10 µL of MgSO4 10 mM were mixed by vortexing. Then, the mixture was incubated at 28 ℃ for 20 min. Following the incubation, the mixture was combined with 5 mL of soft LB agar with 0.6% (w/v) agar and vortexed. After that, the mixture was poured onto the surface of the LB agar plate with 2% (w/v) agar immediately. The agar plate was incubated overnight at 37 ℃. The following day, the visible plaques were counted at the appropriate dilutions, giving between 3 and 300 plaques, then were converted to Plaque Forming Units (PFU) per mililiter [25–28].

$$\frac{{{\rm{PFU}}}}{{{\rm{mL}}}}\,{\rm{ = }}\,{\rm{Plaques}}\,{\rm{counted}}\,{\rm{ \times }}\,\frac{{\rm{1}}}{{{\rm{Dilution}}\,{\rm{factor}}}}\,{\rm{ \times }}\,\frac{{\rm{1}}}{{{\rm{Conversion}}\,{\rm{factor}}}}$$

### The efficiency of plating (EOP)

The EOP assay was carried out to define the potential of each bacteriophage against a variety of target bacteria using the double agar overlay assay. EHEC-nmr was used as the reference bacteria for bacteriophage BI-EHEC, while EPEC-nmr and ETEC-nmr were used as the target bacteria. Meanwhile, EPEC-nmr was used as the reference bacteria for bacteriophage BI-EPEC, while EHEC-nmr and ETEC-nmr were used as the target bacteria. We used EHEC-nmr; EPEC-nmr; and ETEC-nmr isolated from clinical samples provide by US-Namru. The purified bacteriophage was diluted in four dilutions, which were 10^− 6^ to 10^− 9^ dilutions, with sterilized SM buffer. For the double agar overlay assay, 200 µL of the diluted bacteriophage, 200 µL of the mid-log phase target bacterial culture (108 CFU/mL or OD600 = 0.132), 50 µL of CaCl2 10 mM, and 10 µL of MgSO4 10 mM were mixed by vortexing. Then, the mixture was incubated at 28 ℃ for 20 min. Following the incubation, the mixture was combined with 5 mL of soft LB agar with 0.6% (w/v) agar and vortexed. After that, the mixture was poured onto the surface of the LB agar plate with 2% (w/v) agar immediately. The agar plate was incubated overnight at 37 ℃. The following day, the visible plaques were counted at the appropriate dilutions, giving between 3 and 300 plaques. Titer determination was performed for each positive result of the host bacteria. The EOP value was calculated by dividing the average PFU on the target bacteria by the average PFU on the host bacteria. The EOP value was categorized as high efficiency (0.5-1), medium efficiency (0.2–0.5), low efficiency (0.001-0.2), and not effective (0–0) [7].

### Bacteriophage application on Food samples

Several food samples, such as tomato, lettuce, milk, and chicken skin, were purchased from the local market in Tangerang, Indonesia. Fresh tomatoes and fresh lettuce were rinsed with tap water and swabbed with 70% ethanol for 3 min on the surfaces to decontaminate the samples and eliminate bacteria. After that, the samples were cut into pieces (approximately 2 cm^2^ or around 1 gram) with a sterile knife or scalpel. Then, both samples were exposed to UV light under laminar airflow (ESCO) for about 30 min with the distance around 30 cm to ensure the killing of any possible bacteria contamination. Following the exposure to UV light, each sample was placed into a sterile 50 mL Falcon tube (Corning®). Around 5 mL of milk was placed in a 15 mL sterile Falcon tube (Corning®). Then, the Falcon tubes were sterilized in an autoclave for 15 min at 121 ℃. The chicken skin was rinsed with tap water and cut into pieces (approximately 2 cm^2^ or around 1 gram), which were placed in 50 mL of Falcon tubes (Corning®). Then, the Falcon tubes were sterilized in an autoclave for 15 min at 121 ℃. EHEC was used as the reference bacteria for the bacteriophage BI-EHEC treatment on the sample, while EPEC was used as the reference bacteria for the bacteriophage BI-EPEC treatment on the sample. Following the sterilization, all the sterilized samples were inoculated with 100 µL of the mid-log phase host bacteria culture and were incubated at 28 ℃ for 45 min. Following the incubation, the samples were combined with 100 µL of the purified bacteriophage lysate at a Multiplicity of Infection (MOI) of 100. For the negative control, the samples were treated only with 100 µL of the bacteriophage lysate. For the positive control, the samples were treated only with 100 µL of the mid-log phase host bacteria culture. The samples were incubated at 4 ℃ for 1 day and 6 days. Following the incubation, the samples were mixed with 10 mL of SM buffer and vortexed. Then, the mixture was serially diluted up to 10 − 3 of dilution with sterilized SM buffer. Each dilution was spread onto the LB agar with a volume of 100 µL for each dilution. Then, the agar plates were incubated overnight at 37 ℃. The following day, the viable bacterial count was calculated by determining the colony forming units per milliliter (CFU/mL) [17, 29–34].

### Statistical analysis

The statistical analysis was performed for the bacteriophage application on the food samples by a one-way ANOVA and Tukey’s-B test, in which the level of difference was determined at p ≤ 0.05. Meanwhile, the control and treatment pairing of each sample was performed with paired-sample T-Tests to determine if they were significantly reduced or not with a level of difference at p ≤ 0.05 [35].

## Results

### Bacteriophage titer

The titer values of bacteriophage BI-EHEC and BI-EPEC were determined using the double agar overlay assay. Bacteriophage BI-EHEC infected the EHEC pathogenic bacteria as a reference strain, while bacteriophage BI-EPEC infected the EPEC pathogenic bacteria as a reference strain. Both results were proven by the appearance of clear and circle plaques (supplementary Fig. 1). In this study, the titer of BI-EHEC was 9.31 ± 4.6 × 10^9^ PFU/mL, while BI-EPEC was 1.22 ± 3.8 × 10^10^.

### EOP Assessment

The EOP was carried out to identify the host range of each bacteriophage used in this research. The host range of bacteriophage BI-EHEC of EHEC was determined against EPEC and ETEC as the host pathogenic bacteria, while bacteriophage BI-EPEC was treated against EHEC and ETEC as the host pathogenic bacteria.

Bacteriophage BI-EHEC showed low efficiency toward EPEC but high efficiency toward ETEC, while bacteriophage BI-EPEC showed high efficiency toward both EHEC and ETEC (Table 1).

**Table 1:** Efficiency of Plating (EOP) Value for BI-EHEC and BI-EPEC.

Bacteriophage	Efficiency of Plating		
	EHEC	EPEC	ETEC
BI-EHEC	**1.00**	0.10 ± 0.04	2.95 ± 0.51
BI-EPEC	1.10 ± 0.19	**1.00**	1.21 ± 0.23

Data in the table above were shown in mean ± standard error

### Bacteriophage application on Food samples

The effectiveness of each bacteriophage was determined by calculating the bacterial reduction (total plate count) after artificial contamination of the host pathogenic bacteria in various food samples, including tomato, lettuce, milk, and chicken skin. The incubation times were 1 day and 6 days, and both treatments were incubated at 4 $?$ temperature. The results showed that the number of host pathogenic bacteria in each food sample with the different matrices were significantly reduced for both treatments (1 day or 6 days of incubation time) (Table 2).

**Table 2:** Application of Bacteriophage BI-EHEC and Bacteriophage BI-EPEC on Various Food Samples for 1-Day and 6-Days of Incubation time

Phage	Time of Incubation (Days)	Samples	Control (CFU/mL)	Bacteriophage Treatment (CFU/mL)	Bacteria Reduction (log)	Bacteria Reduction (%)
	1	Tomato	1.13 ± 2.78 × 10^5a^	9.59 ± 1.89 × 10^3a*^	1.07 ± 0.002	91.51 ± 0.04
BI-EHEC		Lettuce	7.25 ± 2.78 × 10^5d^	5.45 ± 2.93 × 10^4d*^	1.12 ± 0.01	92.47 ± 0.18
		Milk	1.79 ± 2.42 × 10^5b^	3.13 ± 3.16 × 10^4c*^	0.75 ± 0.009	82.48 ± 0.36
		Chicken Skin	2.72 ± 3.21 × 10^5c^	2.63 ± 3.95 × 10^4b*^	1.01 ± 0.03	90.31 ± 0.57
	6	Tomato	1.89 ± 2.86 × 10^4a^	2.26 ± 2.58 × 10^3a*^	0.92 ± 0.002	88.03 ± 0.05
		Lettuce	1.43 ± 2.90 × 10^5b^	2.73 ± 2.42 × 10^3a*^	1.71 ± 0.009	98.09 ± 0.04
		Milk	1.65 ± 3.09 × 10^5d^	9.66 ± 3.08 × 10^4b*^	0.23 ± 0.002	41.50 ± 0.28
		Chicken Skin	1.46 ± 4.16 × 10^5c^	1.06 ± 3.43 × 10^5c*^	0.13 ± 0.001	27.37 ± 0.21
BI-EPEC	1	Tomato	3.64 ± 3.55 × 10^5b^	1.10 ± 2.24 × 10^5c*^	0.42 ± 0.09	69.82 ± 0.92
		Lettuce	1.24 ± 2.93 × 10^5a^	3.09 ± 2.09 × 10^3a*^	1.60 ± 0.01	97.49 ± 0.06
		Milk	9.71 ± 4.80 × 10^5d^	1.23 ± 2.53 × 10^5d*^	0.89 ± 0.006	87.29 ± 0.17
		Chicken Skin	4.43 ± 3.99 × 10^5c^	2.54 ± 2.91 × 10^4b*^	1.24 ± 0.02	94.24 ± 0.24
	6	Tomato	9.02 ± 3.70 × 10^4a^	3.56 ± 3.08 × 10^3a*^	1.40 ± 0.01	96.05 ± 0.09
		Lettuce	1.68 ± 3.10 × 10^5d^	8.04 ± 3.36 × 10^4d*^	0.33 ± 0.01	53.44 ± 1.14
		Milk	1.37 ± 4.19 × 10^5b^	5.08 ± 3.63 × 10^4c*^	0.42 ± 0.001	62.77 ± 0.11
		Chicken Skin	1.51 ± 4.29 × 10^5c^	4.55 ± 3.68 × 10^4b*^	0.52 ± 0.009	69.91 ± 0.65

Data were shown in mean ± standard error value, different letters in each column indicated significant differences, α ≤ 0.05. “*”: shown significant differences between control and bacteriophage treatment for each food sample

## Discussion

In this study bacteriophage BI-EHEC and BI-EPEC from previous study were applied as biocontrol agent to control EPEC and EHEC which were artificially contaminated on various food samples. BI-EHEC reduced the number of EHEC with bacterial reduction value above 0.13 log_10_, while BI-EPEC reduced number of EPEC with reduction value above 0.33 log_10_. A plaque assay was used to determine the concentration of infectious phage particles, in which the dilutions of the phage were combined with a specific host bacterium and dispersed evenly onto a double agar overlay or soft agar overlay medium. Following the incubation on the agar plate, the host bacteria formed a lawn on the solid medium, except for the area where the infectious phage particles lysed or inhibited the growth of the host cells, which could be seen with the naked eye as a localized clear or translucent circle zone called a plaque in the top agar [36]. Double overlay agar provides bacteria host cells with nutrients and a growth medium that is fluid enough to allow them to form a confluent lawn structure. Meanwhile, bacteria host cells that are infected with the lytic bacteriophage before the solidification of agar allows the phages to replicate within the cell and produce progeny phages that are visible as clear circles form called plaque [37].

The use of MgSO_4_ and CaCl_2_ has a major impact on plaque formation. The addition of the nutrients makes the plaques more uniform and clear up when the concentration of the nutrients is about 25 mM, but then becomes more turbid and irregular in size at higher concentrations. The presence of Ca^2+^ and Mg^2+^ cations (divalent metal ions) improves the efficiency of phage to lyse its bacterial host cell by increasing the adsorption rate of the bacteriophage to infect the host cell, also making the plaque formation larger [20].

Bacteriophages can be grouped into the lytic phase and the lysogenic phase. The lytic bacteriophage is capable of killing or lysing the host cell, while the lysogenic bacteriophage integrates and stays as part of the genome of the host cell for a time, replicating along with the bacterial host cell genome for a time. When special environmental conditions happen (a stress condition), lysogenic bacteriophage will cut off the phage genome from the bacterial host cell DNA, pack it into a protein shell, and the mature phage will lyse the host bacteria cells. As a biocontrol agent, the application of bacteriophage in controlling foodborne disease requires lytic bacteriophage that can lyse the host cell directly, while lysogenic bacteriophage can lead to unwanted gene transfer [38].

In a previous study [22], bacteriophage BI-EHEC had a titer value of about 1.63 ± 0.46 × 10^10^ PFU/mL, while bacteriophage BI-EPEC had a titer value of about 2.62 ± 0.67 × 10^10^ PFU/mL. The titer concentration of these bacteriophages can drop because of the long-term storage in cold temperatures, which is related to the cryoprotectant agent that had been used (glycerol). This cryoprotectant works by retaining water within the cell, preventing excessive dehydration due to exposure to concentrate solutions [39]. Due to the storage in cold temperatures, viability loss can happen due to damage to the cytoplasmic membrane or different structural and functional macromolecules in the cells. Several studies demonstrate that storage of phage at low temperatures (below 4 $?$) reduces phage activity significantly. This reduction could be performed to test the effect that freezing or thawing may have on phage ultrastructure, which is commonly relevant to tailed phages from the *Myoviridae* family. Delicate phage tail and tail fibers can become dissociated from the virus head due to changes in osmotic pressure, which makes the phage ineffective as a control agent [40]. Moreover, a decrease in the phage titer concentration can be caused by the crystal structure of the ice that forms during long-term storage at low temperatures, which may destroy the phage, even if glycerol was added [41].

Bacteriophage BI-EHEC of EHEC and bacteriophage BI-EPEC of EPEC lysed another pathogenic bacterial cell with different EOP values (Table 1). Based on the results, bacteriophage BI-EHEC of EHEC would have a higher potential to attack ETEC than EPEC. Moreover, ETEC also had a higher preference for bacteriophage BI-EPEC of EPEC because its EOP value was much higher than EHEC. Pathogenic bacteria, such as EHEC, EPEC, and ETEC, are Gram-negative bacteria that have lipopolysaccharides (LPS) on their outer membranes, which are well-known receptors of RBP. LPS is composed of three parts, which are lipid A, core polysaccharide, and O-antigen [42]. The difference in the ability of bacteriophages to infect Gram-negative bacteria could be caused by differences in the O-antigen of LPS. Bacteriophages recognize the O-antigen with their tail fibers or tail spike proteins, which hydrolyze the O-antigen to initiate penetration of the tail. Besides LPS, bacteriophages also can attach to the outer membrane protein of *E. coli* cells, which are OmpC and OmpF [17]. When OmpC is available, the phage can infect the terminal sugar residue of LPS, but when OmpC is absent, the phage can attach to LPS chains with an exposed terminal glucose residue [43]. *Myoviridae* phages, which are BI-EHEC and BI-EPEC, the kinked lateral tail fibers contact primary receptor are usually OmpC on the bacterial cell surface but when OmpC is not available it can be sugar motif in the LPS [44].

Bacteriophages BI-EHEC and BI-EPEC were particularly effective at reducing the population of EHEC and EPEC, which were artificially contaminated in tomato, lettuce, milk, and chicken skin, and incubated at low temperature (4 $?$ ) for 1 day or 6 days. This might be due to the high stability of bacteriophages and the reduced growth rate of their bacterial hosts at this low temperature [45]. The adsorption of phage is affected by the food matrices. In this study, we used tomato (solid; smooth), lettuce (solid; rough), milk (liquid), and chicken skin (solid; complex). Different foods contain different structures, chemical compositions, and nutrition which could affect the phage adsorption process to the host pathogenic bacteria [46]. In this research, the multiplicity of infection (MOI) between the phage and bacteria host cell was established at 100, where the adsorption of phage was allowed to continue to completion, also phage can adsorb randomly to susceptible cells [29]. Food matrix components, such as soluble protein, amino acids, and sugars, as well dried food may affect the activity of phage [47].

Based on the results in Table 2, bacteriophage BI-EHEC, after 1 and 6 days of incubation time, showed the highest percentage of bacterial reduction on lettuce. Moreover, bacteriophage BI-EPEC, after 1 day of incubation time, showed the highest percentage of bacterial reduction in lettuce, while at 6 days, it was highest in tomato. In solid samples with a smooth surface, the presence of the sample fluid (such as tomato juice from the inner flesh) can increase the ability of the phage suspension distribution to attach and infect the host cell. The host pathogenic bacteria liquid suspension was inoculated immediately to the fresh cut of tomato and lettuce to stimulate the pathogen to grow rapidly in a nutrient-dense environment on the samples before the samples dried. Enteric pathogens can adapt to plant surfaces and access the nutrients in the exudates released from the plants [48]. Liquid samples are considered easier for bacteriophage to infect and reduce the host pathogenic bacteria because the phage suspensions can diffuse freely in the samples [48]. However, the percentage of bacterial reduction of BI-EHEC and BI-EPEC in milk was not as high as in the other samples, either after 1 day or 6 days of incubation. Some components in milk have antiviral activity, such as lactoferrin, which can inhibit the adsorption of phage to bacteria cells. In addition, the presence of immunoglobulins covers up the bacterial cell surface, which can reduce the adsorption of bacteriophages to the host cell [49].

Food samples with solid and uneven surface areas, such as chicken skin, are the most difficult samples to be treated with phage. Both bacteriophage BI-EHEC and bacteriophage BI-EPEC had a low percentage of bacterial reduction on chicken skin at both 1 day and 6 days of incubation. This can be caused by the matrix of the chicken skin. Thus, food samples that contain large surface areas and certain material compositions, such as a high fat content and feather follicles, might serve as a refuge for bacteria [45]. The structure of chicken skin is accompanied by feather follicles and folds on the chicken skin surface along with the oils and fats, which have the potential to trap pathogenic bacteria and make the bacteriophages unable to attach to the pathogenic bacteria [50].

For the bacteriophage to be applied as biocontrol, it is necessary to determine their genomic properties. One of the phage that we used which is BI EHEC have been characterized through genetic analysis, and it was found that there were no virulence properties, antibiotic resistance genes as well as lysogenic protein among annotated genes which implied BI-EHEC a lytic life cycle [51].

## Conclusions

In addition to infecting the host bacteria (EHEC and EPEC), bacteriophages BI-EHEC and BI-EPEC, used in this research, also infected other pathogenic bacteria with varied efficiencies. Bacteriophage BI-EHEC and bacteriophage BI-EPEC reduced the population of their respective host pathogenic bacteria with a variated percentage of bacteria reduction on various food surfaces in different periods and storage conditions. Both phages show the potential to be used as biocontrol agents.

### Limitations

The food samples is still limited, it is important to continue assay the application in increase the variation of food samples. The EOP assay also limited to several bacteria, it is need to be tested to various food borne pathogenic bacteria. We used two kind of bacteriophage, one of them BI-EHEC the genomic properties have been characterized, the other one BI-EPEC have not been assessed through DNA sequencing analysis.

## Electronic supplementary material

Below is the link to the electronic supplementary material.

Supplementary Material 1

## Data Availability

The datasets used and/or analysed during the current study are available from the corresponding author on reasonable request.

## Abbreviations

EOP: Efficiency of Plating
FBD: Foodborne disease
CFU: Colony Forming Unit
PFU: Plaque Forming Unit
RBP: Receptor Binding Protein
Omp: Outer Membrane Porins
