# Supplementary material for: Application of BI-EHEC and BI-EPEC bacteriophages to control enterohemorrhagic and enteropathogenic escherichia coli on various food surfaces
Source: BMC Res Notes. 2023 Jun 13;16:102. doi: 10.1186/s13104-023-06371-6 (PMC10262513; doi:10.1186/s13104-023-06371-6)
Supplement: Supplementary file 1 — Supplementary Material 1 [file 13104_2023_6371_MOESM1_ESM.docx]

SUPPLEMANTARY FILE


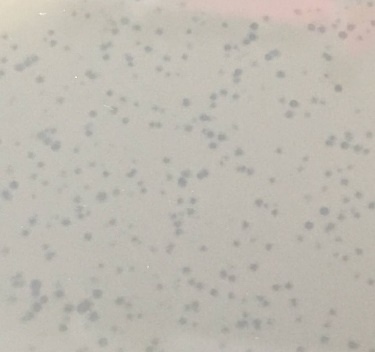

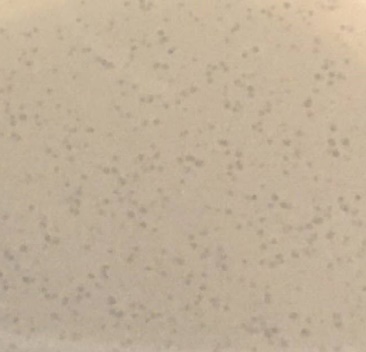


Supplementary Figure 1. Clear zone and circle plaques of bacteriophage BI-EHEC with EHEC

pathogenic bacteria as the host cell (left) and bacteriophage BI-EPEC

with EPEC pathogenic bacteria as the host cell (right).
